# Hybrid Angio-CT with DICI-CT in Interventional Oncology and Beyond: A Narrative Review

**DOI:** 10.3390/cancers17193116

**Published:** 2025-09-25

**Authors:** Michael Moche, Arjen Bogaards, Andreas Horst Mahnken, Philipp Paprottka, Jonathan Nadjiri, Maciej Pech, Thierry de Baere, Bruno Calazans Odisio

**Affiliations:** 1 Department of Interventional Radiology, Heart Center & Helios Park-Hospital, 04289 Leipzig, Germany; 2 Siemens Healthineers, 91301 Forchheim, Germany; 3 Department of Diagnostic and Interventional Radiology and Nuclear Medicine, St. Josef Hospital, Ruhr University Hospital Bochum, 44791 Bochum, Germany; 4 Department of Interventional Radiology Clinic Rechts der Isar, Technical University Munich, 80333 Munich, Germany; 5 Department of Radiology and Nuclear Medicine, University Clinic Magdeburg, 39112 Magdeburg, Germany; 6 Department of Interventional Radiology, Gustave Roussy, 94800 Villejuif, France; 7 Department of Interventional Radiology, The University of Texas MD Anderson Cancer Center, Houston, TX 77030, USA

**Keywords:** Image-guidance, Angio-CT, hybrid, multi-modality minimally invasive, intra-arterial CT, interventional oncology, acute care

## Abstract

Background/Objectives: Hybrid Angio-CT suites have emerged in response to the growing demands for innovation and procedural complexity in minimally invasive therapies. It is hypothesized that enhanced image guidance capabilities enabled by multimodality imaging can improve procedural safety, accuracy, and efficacy. However, due to the current lack of sufficient data to support a systematic review, the objective of this article is to present a comprehensive synthesis of the existing literature through a narrative review. Methods: This narrative review is based on purposefully identified research reports, their critical evaluation, and synthesis by a group of experienced users. The analysis covers three key areas: (1) current state of available technologies and functionalities, (2) novel perspectives through ‘Direct Intravascular Contrast media Injection CT’ (DICI-CT), and (3) the role of Angio-CT in established and emerging image-guided procedures. Results: The review presents typical configurations and room layouts for Angio-CT systems and discusses further technological improvement potential. Selected literature is complemented by expert experience to report on the current state of the art and demonstrate its use and efficiency. Based on our expert experience, it is demonstrated how DICI-CT can be used to reduce contrast dose and improve lesion visualization, targeting, and endpoint determination. Furthermore, in this review the advantages, including survival benefit (i.e., in trans-arterial chemoembolization and in blunt trauma) and cost-effectiveness (i.e., in emergency care), are reviewed with reference to oncologic and non-oncologic applications in both elective and emergency medicine. Conclusions: Hybrid Angio-CT suites can provide significant additional imaging information with the potential to improve image-guided procedures. This perspective is increasingly supported by retrospective data in interventional oncology and beyond. Provided that further technological advancements are achieved and prospective clinical data substantiates the anticipated clinical and economical benefits, hybrid Angio-CT suites are anticipated to play a key role in the multimodality interventional suite of the future.

## Simple Summary

Hybrid Angio-CT systems combine the two foundational imaging modalities of interventional radiology, fluoroscopic angiography (Angio), and computed tomography (CT) for image-guided therapy. These multimodality suites have been in clinical use for over 25 years, offering synergistic benefits by leveraging the strengths of each modality. Initially grown in interventional oncology, the combination of Angio and CT guidance has since enabled a wide range of complex and innovative applications in other fields. An underestimated core advantage lies in the combined use of angiographic fluoroscopy to allow selective transcatheter administration of contrast agent to the target blood vessel for CT-imaging (Direct Intravascular Contrast media Injection CT—DICI-CT). This can improve the planning, navigation and control of minimally invasive procedures. This narrative review provides an overview of the current knowledge of Angio-CT technology, its current clinical relevance, and emerging future applications, based on a subjective selection of the literature combined with the expert opinion of the authors.

## 1. Introduction

In image-guided therapy, the safety, efficacy, and efficiency of the procedure are highly dependent on the availability and quality of intraprocedural imaging data. The most commonly used imaging modalities, computed tomography (CT) and fluoroscopic angiography (Angio), each have their strengths and limitations. CT is characterized by high soft tissue contrast in large volume 3D cross-sectional images, but with lower spatial and temporal resolution. In contrast, the strength of Angio is real-time imaging with high spatial resolution in 2D projection, but inferior soft tissue contrast. By combining the two modalities, their strengths can be enhanced and their weaknesses compensated in order to maximize image information, especially through Direct Intravascular Contrast Media Injection CT (DICI-CT). For this reason, high-end multimodality interventional suites have gained considerable attention and momentum in recent years, and various concepts have been proposed [1,2,3,4]. At present, however, there is limited consensus on equipment standardization, and many hybrid Angio-CT suites are tailored to the specific requirements of individual clinicians covering a wide range of clinical use cases. This article discusses the combination of the two main “workhorses” of interventional radiology. It is aimed toward facilities that intend to integrate Angio and CT into a multimodality solution, rather than using them as two separate devices.

This narrative review provides a synthesis of the current knowledge on Angio-CT technology, its current clinical importance, and its potential for innovative future applications based on a subjective selection of the literature, combined with the authors’ expert opinion.

## 2. Methods and Technology

### 2.1. Literature Selection Methodology

A comprehensive search of the PubMed database was conducted, covering the period from 1985 to August 2025. The primary search terms included “Angio-CT” (777 results), “Hybrid Angio-CT” (33 results), and “Intra-arterial CT” (17 results). Many of these results were contextually irrelevant, often focusing exclusively on diagnostic CT or conventional angiography without addressing the integrated use of both modalities in interventional settings.

Following manual screening and the inclusion of additional references identified through the authors’ subject matter expertise and familiarity with the field, a total of 83 references were selected for this review. Only studies relevant to the integrated use of angiography and CT and/or DICI-CT were retained.

This methodology combines broad literature coverage with expert insight, appropriate for a narrative review. The inherent limitations—such as reduced reproducibility, subjectivity in selection, and potential bias—are acknowledged and discussed in the Limitations section.

### 2.2. Angio-CT Technology Platform

Typically, newer Angio-CT systems integrate a multi-detector CT (MDCT) scanner placed on floor rails and an angiography unit on ceiling rails into a single cohesive platform. The components are arranged “in-line”, so that each can slide over a single patient table. This allows for CT and Angio to be performed on the same table without need to transfer the patient, eliminating the risk of instrument dislodgement, or compromising sterile conditions due to patient transport between separate rooms. Over the past decade Angio-CT systems have started to become more deeply integrated, allowing for a more straightforward workflow, a common and collision-free control of all components, and accurate, registration-free image data overlay. Yet, the current level of integration is still limited, and there is great potential for future improvements to further optimize the ease of use of the combined system.

### 2.3. Different Vendors of Multimodality Systems

There are currently six vendors offering hybrid Angio-CT-MR systems (Table 1). Notably, three new vendors (Neusoft Medical, United Imaging, and Philips Healthcare) started offering such systems only in the last three years. By far, most multimodality installations are Angio-CT combinations mainly used by interventional radiologists.

### 2.4. Utilization and Efficiency

Improved utilization and efficient use of hospital resources is imperative to justify the additional investment in hybrid Angio-CT rooms. Some analyses of oncological and traumatological applications have already shown advantages.

Shinomiya et al. [5] found in 346 patients with HCC that the mean hospitalization period was significantly reduced from 65 to 36 days after introducing an Angio-CT system and advanced catheters for arterial embolization.

Erinjeri et al. [6] described in a total of 3591 procedures that scheduling flexibility increased with the introduction of three Angio-CT systems at a tertiary cancer center, where complex procedures were performed faster, reducing room occupation time for combined embolization and ablation of liver tumor procedures from 300 to 217 min (*p* < 0.01). Feinberg et al. [7] found that replacing an angiography room with a combined Angio-CT system led to a 12% increase in procedure volume in the combined room and a 39% increase in CT scan volumes in the department, possibly explained by specialization. Fergus et al. [8] reported that procedures in a single-room hybrid Angio-CT became more complex, increasing the average reimbursement per case and yearly revenue by 23%, vs. the previous stand-alone angiography suite, nearly covering the hybrid room’s extra cost within the first year.

Frellesen et al. [9] showed that a dual-room CT solution with shared CT on rails and separated with a sliding door generated the same throughput as two separate CT units with the same workforce.

The authors of a health technology assessment concluded that the use of an Angio-CT system not only prolonged survival but was also likely cost-effective in Japan for the treatment of major trauma patients compared with a conventional emergency department [10]. They showed a gain of 1.03 quality-adjusted life years (QALYs) and an incremental cost-effectiveness ratio (ICER) of $32,522 per QALY, below the Japanese willingness-to-pay threshold of $47,619 per QALY.

## 3. Imaging Enabled by Angio-CT

### 3.1. Computed Tomography with Direct Intravascular Contrast Media Injection (DICI-CT)

DICI-CT refers to CT imaging with contrast medium (CM) administered directly into the target vessel via an intravascular catheter placed under fluoroscopic guidance. This approach is enabled by hybrid Angio-CT systems and eliminates the need for patient transfer between imaging modalities. Although DICI-CT is not a novel concept, it remains an underutilized and underappreciated capability of hybrid Angio-CT platforms. In contrast to conventional contrast-enhanced CT (ceCT), where CM is injected into a peripheral vein and diluted through systemic circulation, DICI-CT allows for localized contrast delivery, resulting in enhanced visualization of the region of interest. In this section, we present a synthesis of the reported advantages of DICI-CT as documented in the literature, complemented by our expert interpretation and clinical experience. Where applicable, we also provide practical recommendations and share best practices derived from our expert user group in effort to support broader adoption. To avoid beam hardening artifacts, CM must be highly diluted (1:5 to 1:10 CM to saline ratio), adjusted according to injection speed. While DICI-CT can be performed using a conventional angiography unit followed by transfer to a separate CT room [11,12,13], this carries risks of catheter dislodgement and contamination. Compared to ceCT, DICI-CT significantly reduces CM volume, making it suitable for patients with impaired renal function. For this purpose, DICI-CT has been utilized in EVAR procedures [14] and extremity CTA [15,16], with limited CM volume. In procedures requiring repeated ceCTs, such as intraprocedural controls during complex tumor ablations, DICI-CT allows more single injections before reaching critical CM thresholds. It also provides superior local tissue contrast, enhancing sensitivity for detecting small hypervascular liver lesions like HCC or residual tumors (see also Section 3.2). In animal models, DICI-CT enabled visualization of vascular structures smaller than 1 mm, such as perforator arteries [17].

DICI-CT requires a high-pressure dual-head injector due to rapid phase separation in diluted CM. Single-head injectors, commonly used in angiography, result in uneven contrast—initially too high, followed by complete absence. Planning DICI-CT involves adjusting scan parameters, especially pitch, to synchronize with the CM bolus. For intra-aortic injections, it is critical to ensure the scan does not overtake the bolus. Angiographic determination of bolus flow velocity is recommended. Other parameters—dilution ratio, injection volume, and speed—must be tailored individually. See injection protocol recommendations in Table 2 based on authors’ experience due to lack of reliable data.

As a relatively new technique and absence of standardized protocols, implementation requires a prolonged learning curve. In super-selective DICI-CT, used for imaging circumscribed organ areas prior to targeted embolization, injection speed must be precisely determined via prior DSA with manual CM injection to avoid reflux and inaccurate enhancement.

For CT-guided punctures, typically native single-slice imaging or CT fluoroscopy is used. DICI-CT enables the acquisition of contrast-enhanced images. Due to the low CM requirement, repeated bolus injections can be performed simultaneously with imaging, providing reliable visualization of previously invisible vascular structures or development of the ablation area under local thermal treatment (see also Section 3.3 and Section 3.4).

### 3.2. DICI-CT for Superior Lesion Visualization

For over 35 years, primarily Japanese studies have demonstrated that arterial DICI-CT with low CM volumes provides superior tumor visualization in the liver. Matsui et al. (1985) showed that CT arterial portography (CTAP) via the superior mesenteric artery had high sensitivity for small HCC [18]. Murakami et al. (1997) further improved sensitivity by combining CTAP with CT hepatic arteriography (CTHA) [19], which also applies to colorectal liver metastases [20].

DICI-CT has shown higher sensitivity than ceCT, ceMRI, and ceUS for detecting small insulinomas [21,22] and hypovascular tumors such as previously undetected colorectal liver metastases. CTHA-CTAP or CTHA alone outperformed conventional imaging and 18 F-FDG PET CT [20,23], enabling earlier and more reliable detection—critical for treatment planning [23,24].

Compared to cone-beam CT (CBCT) with intra-arterial CM, DICI-CT has demonstrated superior tumor identification in the liver [25]. Table 3 outlines key differences (mainly based on authors’ experience due to lack of reliable data) between CBCT and conventional CT that contribute to DICI-CT’s higher sensitivity.

### 3.3. DICI-CT for Enhanced Percutaneous Targeting

**Figure 1:**
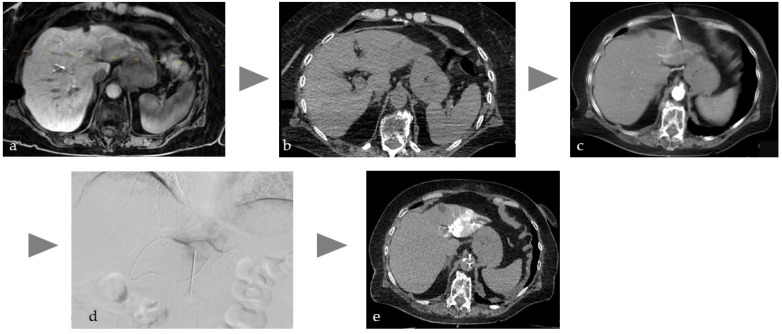
Illustration of the workflow of a combined intervention with percutaneous CT-guided biopsy and immediate transarterial chemoembolization of a cholangiocellular carcinoma in the left lobe of the liver in a patient on anticoagulation due to atrial fibrillation. (**a**) planning MRI (T1w late phase after liver-specific contrast agent) with tumor in the left lobe of the liver, (**b**) native planning CT with weakly hypodense tumor, (**c**) needle puncture with single slice CT and intra-aortic CM injection (15 mL volume with 1:10 dilution corresponding to about 1.5 mL BM), (**d**) DSA with super-selective probing of the feeders to segment 2 and placed needle to perform embolization directly after biopsy to avoid bleeding, (**e**) single slice CT image after lipiodol embolization of the tumor with no evidence of active bleeding.

Manual needle placement under single-slice or fluoroscopic CT imaging benefits from synchronized injection of diluted CM via a dual-head injector, overcoming the limitations of native CT guidance (Figure 1). This improves safety and accuracy, especially in multi-needle procedures near complex vascular and organ structures, such as the pancreas. Given the prevalence of vascular complications in pancreatic tumor ablations, DICI-CT may prevent vascular injury and enable immediate endovascular treatment if needed in case of complications [12,13].

Robotic or software-assisted needle navigation requires two high-quality CT scans—one for planning and one for verification. Intra-arterial DICI-CT provides this image quality and, due to minimal CM use, allows multiple scans with reduced renal risk.

### 3.4. DICI-CT for Therapeutic Endpoint Determination

DICI-CT’s imaging capabilities support quantitative assessment of therapeutic endpoints in transarterial and percutaneous procedures. Selective DICI-CT enables precise intraprocedural quantification of affected tissue volume, as demonstrated in partial splenic embolization [27].

CT’s superior soft tissue contrast, spatial resolution, and quantitative capabilities (e.g., Hounsfield units) make it ideal for monitoring tissue changes during interventional oncology procedures. Compared to MR imaging, CT is more accessible and easier to use.

Taiji et al. [28] showed that enhancement mapping from intraprocedural CTHA—a DICI-CT application—accurately predicted initial response in HCC patients undergoing TACE. Their method achieved 94.7% sensitivity (95% CI, 74.0–99.9) and 100% specificity (95% CI, 87.2–100) at first imaging follow-up (median 6.7 weeks). Feeding arteries to residual enhancement areas were identified in all 18 HCCs with non-complete response, guiding further embolization. This highlights the limitations of DSA-based endpoint assessment and the potential of DICI-CT.

Repeated CTHA during thermal ablation can improve local tumor control compared to conventional CT-guided ablation of colorectal liver metastases [29,30]. The low volume of highly diluted CM allows repeated imaging with negligible renal toxicity. The impact of CTHA on incidental metastasis detection, procedural planning, and ablation margin assessment is being evaluated in the Phase 2 STEREOLAB trial (NCT05361551) [31].

Selective tumor tagging with lipiodol [32,33], which is highly visible and persistent in CT, facilitates safety margin evaluation during control scans. Clinical data over two decades support transarterial embolization prior to percutaneous ablation for liver and kidney tumors, especially for larger tumors [34,35,36,37,38,39,40]. For HCC >3 cm, this data led to German guidelines strongly recommending TACE before thermal ablation [41], though this is not yet universally adopted.

Angio-CT enables treatments to be performed in a single session, combining angiographic catheter navigation with CT-guided ablation. The ability to repeat contrast media injections allows for immediate evaluation of ablation success and the early detection of potential complications. Importantly, the integrated imaging environment facilitates prompt intervention should complications arise, potentially enhancing the overall procedural safety.

## 4. Clinical Applications

### 4.1. Interventional Oncology (IO)

The clinical indications for Angio-CT’s cross-sectional capabilities are primarily in body IR and IO interventions including musculoskeletal malignancies.

In the context of interventional oncology, the Angio-CT platform has been reviewed by Tanaka et al. (2014), Yevich et al. (2018), and Taiji et al. (2021) [42,43,44]. These reviews discuss the technical requirements of Angio-CT systems, including room setup, anesthesia, patient positioning, and improved imaging performance compared to regular CBCT, with their specific imaging acquisition techniques. They highlight how Angio-CT vs. using angiography or CT alone optimizes procedures by improving target tumor depiction, targeting, and treatment delivery assessment.

In addition, the authors argue that Angio-CT systems allow interventionalists to safely perform riskier procedures than before due to advantages of multimodality imaging for better planning, agent delivery, assessment of therapeutic success, and immediate rescue in case of complications. Finally, the quantitative capabilities of CT imaging are valuable for determining therapeutic endpoints in image-guide oncological procedures.

Lin et al. [25] compared Angio-CT with DICI-CT to CBCT in oncological interventions and found superior contrast resolution, a larger field of view, fewer artifacts, and the possibility of CT fluoroscopy, though with potentially higher radiation exposure and costs.

A landmark study by Toyoda et al. [45] in 1312 patients evaluated the effects of Angio-CT with DICI-CT in the treatment HCC with TACE and found significantly higher survival rates in patients who underwent TACE with intraprocedural CT than in those treated under angiography alone (*p* < 0.0023), attributing this to improved targeting and avoidance of false embolization.

### 4.2. Wire-Guided Percutaneous Drainage Procedures

Over the last decades, a variety of initially fluoroscopically guided procedures were shown to be either safer or more effective when performed under CT guidance. This includes procedures such as percutaneous gastrostomy in case of unfavorable position of the stomach [46], enterostomy, or nephrostomy in non-dilated renal pelvises [47]. Other potential indications include biliary drainage [48], abscess drainage [49], and extravascular foreign body removal [50]. All these procedures have in common that they require percutaneous access as a first step of the procedure, followed by a procedure that benefits from a greater overview and real-time image control. For example, in nephrostomy, it is easier to puncture a (non-dilated) renal pelvis under CT guidance, while antegrade guidewire and drainage placement are usually much easier to perform under 2D fluoroscopy, as the latter provides a better overview and real-time imaging for optimal device control. The same applies to any other types of drainage procedure. To date, most reports on these kinds of procedures describe the use of a mobile C-arm for fluoroscopy. A dedicated Angio-CT system overcomes most of the limitations, such as restricted patient access, field of view, and radiation protection, while providing several major advantages in C-arm handling and table position movement [51]. This hybrid modality provides the technical basis for further development of all complex procedures that depend on precise CT-guided needle placement followed by real-time guided device manipulation under fluoroscopy.

### 4.3. Radioembolization for Liver Cancer

Radioembolization of liver tumors requires careful planning and image analysis to precisely deposit the target dose and to exclusively treat the target area to ensure safe and effective treatment. There is a clear recommendation for the use of cross-sectional images for the targeting of vessels and the identification of feeder branches to tumors. CBCT has been shown to simplify the procedure, with Angio-CT even being superior to CBCT [52,53,54]. Recent studies have shown that Angio-CT with DICI-CT has many advantages, including increased sensitivity for detecting aberrant enteric feeders and satellite lesions, improved detection, and identification of parasitic extrahepatic arteries in HCC, and reduced risk of non-target embolization [55]. In addition, Angio-CT can facilitate personalized dosimetry in patients undergoing SPECT and improve dose calculations for tumors with multiple feeders from different arteries or for radioembolizations performed in multiple steps with targets in close proximity [56]. Overall, in our experience, Angio-CT with DICI-CT may provide radiologists and nuclear medicine physicians with greater confidence in the final catheter position for embolization and a more accurate analysis of the perfused fraction of the tumor, leading to improved treatment outcomes.

### 4.4. Transjugular Intrahepatic Portosystemic Shunt Creation (TIPS)

Emergency TIPS creation outside of regular working hours is associated with reduced outcomes due to the poor clinical status of the patients and sometimes due to the lack of experienced sonographers, despite strong recommendations. Consequently, fluoroscopy is often utilized as the solitary modality for image guidance. To overcome this problem, previous studies have evaluated image fusion based on the registration of 3D data from previous examinations, including MR and CT, with the interventional fluoroscopy images [57]. However, this method has pitfalls, such as the impairment of image co-registration due to different body postures and anatomical structures. A recent study demonstrated the feasibility of TIPS creation by a single interventionalist using Angio-CT, which reduced procedure time and radiation exposure while ensuring higher patient safety compared to fluoroscopic guidance alone [58].

### 4.5. Prostate Artery Embolization (PAE) in Benign Prostate Hyperplasia (BPH)

**Figure 2:**
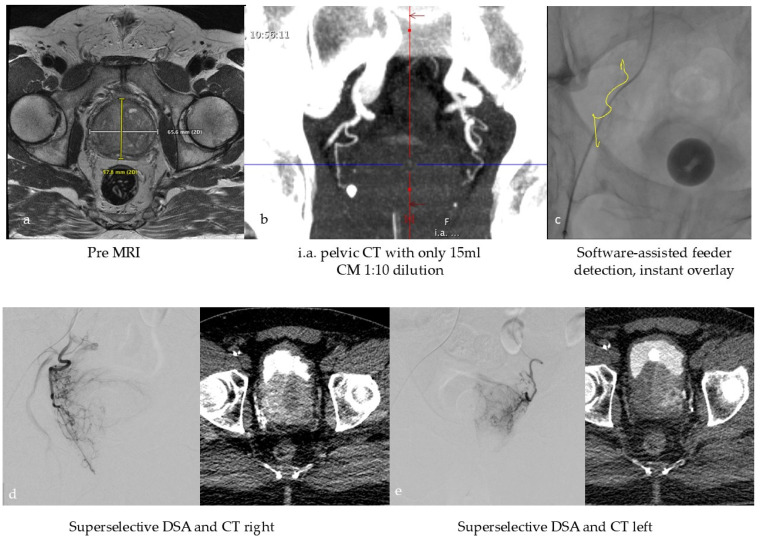
Typical workflow for prostatic artery embolization in benign prostatic hyperplasia (BPH): (**a**) optional pre-interventional MRI to visualize the hyperplasia and exclude tumors, (**b**) intra-arterial CT angiography with 100 mL volume, with 1:10 dilution corresponding to approx. 10 mL CM at the beginning of the intervention to identify the prostate arteries, (**c**) software-based marking of the prostate artery and overlay on fluoroscopy, (**d**,**e**) After super-selective probing of the right and left prostate artery, DSA and CT were performed to ensure selective targeting of prostate tissue.

In the context of tissue devascularization procedures, the concept of super-selective treatment assumes particular significance in the scope of PAE for the treatment of symptomatic BPH, given the notable proximity of the structures at risk. The success of the treatment depends on the identification of the tiny, highly variable feeding vessels and their collaterals [59,60]. This leads to relatively complex procedures with potentially increased radiation dose to the patient and medical staff. Due to the lack of adequate imaging in the early days of PAE, complications due to ischemia in the rectum, bladder, and penis sometimes occurred, although rarely [60]. Consequently, the current consensus on the imaging workflow involves performing a preprocedural CT or MR angiography followed by an intraprocedural selective CBCT scan of each internal iliac artery to identify the relevant feeding prostate arteries. In the second step, the end-stream route intended for embolization must be verified by an additional distal super-selective CBCT to avoid complications associated with non-targeted embolization [61]. As preoperative cross-sectional imaging is not part of the routine BPH clinical workup, a group of experts recently published an innovative viewpoint to perform PAE without preprocedural CT or MRI but in a single session with high-resolution intraprocedural imaging. The authors of this study hypothesize that an Angio-CT suite will allow for a dedicated one-stop workflow with high soft tissue contrast planning images without motion artifacts, like conventional ceCT, and excellent super-selective imaging for precise targeting [62]. This workflow is illustrated in Figure 2.

### 4.6. Prevention and Percutaneous Treatment of Type 2 Endoleaks

**Figure 3:**
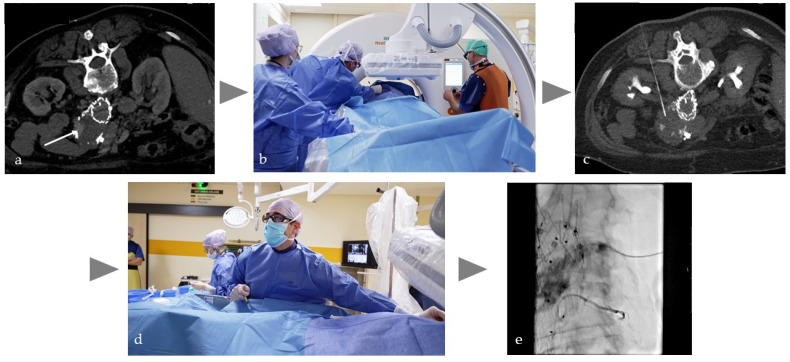
Typical workflow in the percutaneous treatment of a type 2 endoleak after EVAR of an abdominal artery aneurysm. (**a**) Planning CT in prone position with intra-arterial injection of 150 mL volume, with a dilution of 1:10 corresponding to approx. 15 mL CM to detect the endoleak (white arrow) in the aortic sac, (**b**,**c**) translumbar CT-guided fine needle puncture, (**d**) after insertion of the guide wire, placement of the microcatheter under fluoroscopic view, (**e**) controlled embolization of the endoleak with liquid (Onyx18) under fluoroscopic view.

Persistent type 2 endoleaks (pT2EL) after endovascular aortic aneurysm repair (EVAR) occur in up to 29% of cases and are significantly associated with reintervention and fatal aortic rupture [63]. There is clear evidence to support preemptive embolization of relevant aortic side branches (ASB) prior to EVAR, such as larger lumbar and inferior mesenteric arteries, to avoid the development of pT2EL [64,65]. The mean embolization procedure time was reported to be up to 169 min, due to the sometimes challenging localization of multiple small vessels, resulting in a relevant CM amount and radiation dose [66]. The overlay of pre-procedural CT on intraprocedural fluoroscopy is often erroneous and unreliable and requires further development to improve its reliability [67,68], especially to facilitate cannulation of small vessels such as ASB. Although not yet published, the author’s experience shows that intra-procedural intra-aortic DICI-CT and automatic overlay functionality allows an acceptable accuracy of 1 mm for reliable and efficient cannulation of ASB, resulting in a mean fluoroscopy time of less than 25 min. Typically, pT2EL is treated by either endovascular or percutaneous embolization. In the case of percutaneous access in an Angio-CT setting, as illustrated in Figure 3, the first step is intra-aortic dual phase DICI-CT to identify the pT2EL. This is followed by CT-guided percutaneous puncture in the vascularized area in the aortic sac. At this point, embolization with fluids could be performed without sufficient real-time imaging control. However, the endpoint of embolization cannot be precisely determined, which entails the risk of under- or overfilling and consequently under- or overtreatment with corresponding complications. Therefore, this approach is not recommended [69]. It is preferable to secure access by means of a wire and switch to angiography. Under fluoroscopic guidance, a microcatheter is inserted over the wire and the pT2EL can be safely embolized. If necessary, a final CT scan can be performed to assess the outcome of the treatment. Without Angio-CT, the procedure begins with CT-guided percutaneous puncture and wire placement. The patient must then be transferred to the angiography unit in order to perform the proper embolization. This process is extremely inefficient and carries a relevant risk of guidewire dislodgement or contamination of the sterile field. As an alternative single room approach, Rhee et al. [70] reported the use of CBCT in a hybrid OR and image fusion for needle trajectory planning for percutaneous puncture and fluoroscopic embolization without patient transfer.

### 4.7. Emergency One-Stop Strategy: Combined Diagnosis and Treatment

#### 4.7.1. Aortic Aneurysm

**Figure 4:**
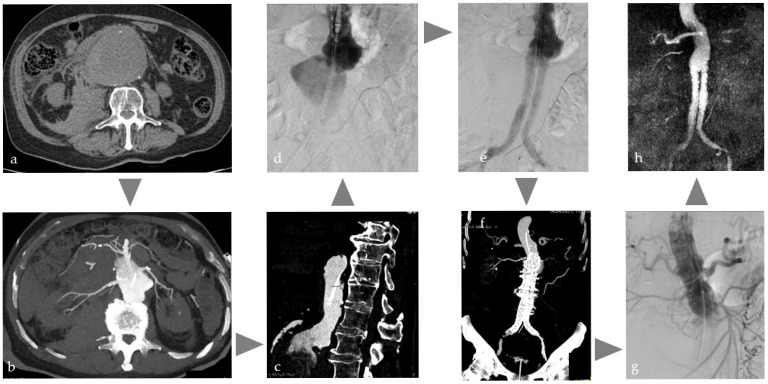
Typical workflow for one-stop treatment of a partially ruptured abdominal aortic aneurysm in a patient with acute abdominal pain and restricted kidney function: (**a**) Due to clinical and sonographic suspicion of a ruptured abdominal aortic aneurysm, native CT on the Angio table to confirm findings, (**b**,**c**) CT with intra-arterial injection of 120 mL volume, with a dilution of 1:5 corresponding to approx. 20 mL CM for planning the EVAR, (**d**) insertion of the main body of the prosthesis, (**e**) control DSA after bi-iliac stent graft with indication of insufficient coverage and type 1a endoleak, (**f**) immediate intraoperative CT control with intra-arterial injection as above to plan prosthesis extension, (**g**) treatment using cuff in chimney technique, (**h**) MRI control the next day with regular findings.

Ruptured aortic aneurysms (rAAA) are associated with high mortality rates and therefore require prompt CT diagnosis and treatment [71]. Depending on the imaging findings, either open surgery or, more frequently, endovascular aortic aneurysm repair (EVAR) is performed. EVAR of rAAA in an Angio-CT unit as one-stop strategy, illustrated in Figure 4, has the exceptional advantage of standard CT imaging but reduced CM administration [14] for diagnosis, treatment planning, and instant intervention; for example, with immediate endovascular placement of an intra-aortic occlusion balloon to control bleeding independent of further treatment decision. If the environment allows automatic image fusion, the CT data can be directly overlaid on the angiographic images without any further registration process to support immediate and precise EVAR. There is one case report that addresses this type of workflow [72]. The Angio-CT features can also be helpful in more complex routine EVAR cases, where the anatomy may be significantly altered by, for example, stiff wires, especially in challenging vessel conditions such as severe vessel kinking due to dilatation. Intraprocedural DICI-CT with immediate overlay may help to better understand changed anatomy. Furthermore, this approach may offer a valuable intraprocedural tool for the detection of certain complications, such as inguinal bleeding, especially in obese patients. Surprisingly, there is no scientific data available on this topic.

#### 4.7.2. Stroke

For many years, endovascular thrombectomy has a class 1a recommendation for selected patients with acute ischemic stroke [73]. As outcome worsens with an increased time window after the event, treatment must be performed as soon as possible after confirming the diagnosis with CT. Pfaff et al. treated 50 patients [74] using a C-arm in a regular CT unit immediately after CT imaging. This resulted in significant reduction in “picture-to-puncture” time with a median of 43 min under full anesthesia and 39 min with conscious sedation vs. 64 min in the stand-alone Angio suite (*p* < 0.0001) with comparable recanalization rates. The limitations of this setting are unaccustomed working conditions with limited radiation protection. Yaeger et al. [75] recommended combination of diagnostics and treatment in an Angio-CT system to reduce that “dead time”. In a recent study enrolling 86 patients by Fujimoto et al. [76] using Angio-CT, the door-to-puncture time (53 vs. 87 min, *p* < 0.001) and door-to-recanalization time (107 vs. 174 min, *p* < 0.001) were significantly shorter than the conventional workflow in this hospital. Analyses during acute and rehabilitation phases showed a shorter median hospital stay in the Angio-CT group (51.5 vs. 95.8 days, *p* = 0.0227) and an 18.3% reduction in mean hospitalization costs compared to the conventional group (*p* = 0.0786).

#### 4.7.3. Trauma Care

Whole-body CT is the globally accepted standard for imaging of life-threatening injuries [77]. It leads to significant survival benefit, especially for patients after blunt trauma requiring emergency bleeding control [1]. First results showed that introduction of an angiography unit into the emergency CT room significantly reduces the time to surgical damage control and transcatheter arterial embolization [1]. Consequently, in Japan, more than 21 trauma centers introduced hybrid emergency rooms with Angio-CT systems for implementation of a new trauma workflow concept [78] which led to the foundation of the “Japanese Association for Hybrid Emergency Room Systems” (HERS). In a retrospective, single-center controlled study by Kinoshita et al. [79], 696 patients were included. Those 336 patients treated in the hybrid Angio-CT group were significantly associated with decreased 28-day mortality and reduced deaths from exsanguination (*p* = 0.001) vs. the 360 patients in the conventional workflow group, probably because of significantly shorter time to CT initiation (*p* < 0.0001) and emergency treatment (*p* < 0.0001). An extended analysis of 1050 patients revealed that the probability of survival improved further in patients with a higher injury severity score [80]. In a study of 96 patients by Ito et al. [81], the time from arrival to control of traumatic pelvic fracture bleeding using embolization was found to be significantly (*p* < 0.01) shorter in the hybrid Angio-CT. A study in 279 patients by Watanabe et al. [82] showed that patients with an injury severity score of ≥16 who were treated using a hybrid Angio-CT approach required a significantly lower amount of blood transfusions when compared to those treated using conventional methods. The global community of trauma care providers is closely observing the recent developments in Japan with great interest. These developments are regarded as representing “the most novel recent advancement in the quest to reduce potentially preventable mortality from traumatic bleeding”. Some observers are pondering if it is time to switch [26], and some already made the switch [83].

## 5. Limitations

The narrative review format was chosen deliberately due to the current lack of sufficient prospective data to support a systematic review. While this format allows for a broader and more flexible exploration of the topic, it inherently introduces limitations. The literature selection was based on purposeful identification of relevant studies by subject matter experts and combined with expert experiences, which, although appropriate for a narrative approach, may introduce selection bias, subjectivity, and limit reproducibility.

Using only two search terms and no inclusion or exclusion criteria further restricts the methodology. The integration of expert opinion, while valuable in contextualizing emerging clinical practices, carries a degree of subjectivity. The review does not adhere to formal standards such as PRISMA (Preferred Reporting Items for Systematic Reviews and Meta-Analysis), is suitable given the narrative nature, but is acknowledged explicitly.

The review relies heavily on retrospective studies and expert experience, which may overstate the perceived benefits of hybrid Angio-CT. While some cited studies offer robust sample sizes, others are smaller, limiting the generalizability of findings. Additionally, the review does not address potential gaps in the literature, such as complication rates, or comparative effectiveness across patient populations.

Although hybrid Angio-CT technology has improved, the current level of integration is still limited, leaving a large potential for future improvements to further optimize the ease of use of the combined system. Despite promising clinical developments, prospective clinical data remain limited.

Furthermore, economic and practical barriers to adoption remain, including high equipment costs, infrastructure requirements, and specialized training needs, which are underexplored.

These factors should be considered when interpreting these findings and projecting the role of hybrid Angio-CT into the future.

To fully realize the clinical and economic potential of Angio-CT, further technological development is required and high-quality studies are essential. Stakeholders—including clinicians, researchers, and industry partners—are encouraged to collaborate to optimize the man–machine interface and generate robust evidence to support broader adoption and standardization.

## 6. Conclusions & Outlook

The aim of this narrative review is to bring together a growing body of emerging but fragmented data on hybrid Angio-CT systems and to provide a consolidated overview based on both the published literature and the practical insights of an expert group actively using these technologies in clinical practice.

The combination of the most important interventional radiology modalities results in more than the sum of the individual advantages and thus increases the handling and comfort of established image-guided treatments such as percutaneous wire-guided procedures. Importantly, it can create synergies that facilitate new procedure combinations such as transarterial embolization and percutaneous ablation in one session with increased accuracy and efficiency. Importantly, it enables standard CT imaging with selective transcatheter contrast media injection into vessels or vascular organ supply (DICI-CT), allowing for unreached tumor visualization, high precision tissue targeting, and previously unimaged therapeutic endpoint determination. The co-registration-free, almost simultaneous fusion of CT and fluoroscopic images in a shared coordinate system with unchanged patient position further enhances spatial precision and procedural efficiency, surpassing traditional overlay techniques based on pre-procedural data. This functionality is one of the reasons why Angio-CT also has relevant applications beyond oncological interventions, e.g., to increase the efficiency of special endovascular procedures. Furthermore, Angio-CT appears to be of great benefit for one-stop emergency strategies, such as in stroke, ruptured aortic aneurysm, or blunt trauma, with the potential to set new worldwide standards for advanced emergency care.

Looking ahead, the field of image-guided therapy is entering a dynamic phase of convergence between imaging and devices. The further integration of emerging modalities such as high-definition angiography, photon-counting CT, low-field MRI, and wireless ultrasound imaging is imminent, promising substantial improvements in image quality, functional information, diagnostic confidence, and procedural planning. Simultaneously, the availability of advanced navigation tools is expanding. Robotic arms and laser-guided systems for percutaneous interventions, as well as catheter robotics for endovascular procedures, are increasingly being paired with intelligent software for needle planning, embolization trajectory guidance, and treatment verification. These developments are expected to enhance the precision and reproducibility of complex interventions. The deeper integration of imaging, navigation, and devices (e.g., locoregional ablation systems) will significantly improve the ergonomics, automation, and decision–support capabilities of hybrid Angio-CT systems. Artificial intelligence and augmented reality are likely to play a pivotal role in this evolution—having the potential to enable real-time image interpretation, predictive analytics, and immersive procedural guidance. Together, these advancements are creating a new ecosystem in which imaging and devices are more tightly interwoven than ever before. This convergence is expected to further improve procedural outcomes, reduce variability, and expand the scope of minimally invasive therapies across a wide range of clinical disciplines.

In conclusion, hybrid Angio-CT carries potential to improve minimally invasive therapy across the entire spectrum of the growing number of image-guided procedures worldwide. Provided that further technological advancements are achieved and prospective clinical data substantiates the anticipated clinical and economic benefits, hybrid Angio-CT is anticipated to play a key role in the multimodality interventional suite of the future.

## Figures and Tables

**Table 1:** Currently available hybrid Angio-CT-MR systems and key characteristics.

Manufacturer	Product Family Name	Room Config	CT Bore Size	CT Cooling	CTP Coverage	4D-CTA Coverage	CTDE	Angio-CT Fusion
United Imaging	uHOR aka Rubik’s Cube Integrated OR	1-, 2-, 3-room Angio-CT and MR	82 cm	Water or Air	4 or 8 cm	4 or 8 cm	Seq.	Manual
Neusoft Medical	NeuAngio-CT, NeuRoboAngioCT	1- and 2-room Angio-CT and endovascular robotics	72 cm	Air	4 or 16 cm	4 or 16 cm	Seq.	Manual
GE Healthcare	INTERACT Angio-CT	1-room Angio-CT	75 cm	Air	4 cm	4 cm	Kvs.	Manual
Philips Healthcare	Philips Angio-CT	1-room Angio-CT	80 cm	Air	8 cm	8 cm	Swd.	Manual
Siemens Healthineers	Nexaris Therapy Suites	1-, 2-, 3-room Angio-CT and MR	82 cm	Water or Air	16.7 cm	30 cm	Twb.	Automated
Canon Medical Systems	Alphenix 4D CT	1-, and 2-room Angio-CT	78, 80, 90 cm	Air	4 or 16 cm	4 or 16 cm	Seq.	Manual

Source data: vendor websites. Abbreviations: Seq.—via two sequential CT scans; Kvs.—via kV switching; Twb.—via twin beam acquisition; Swd.—via sandwich detector; Manual—manual registration and fusion; Automated—instant registration and fusion via a common coordinate system and/or semi-automated via two fluoroscopic acquisitions and 3D registration.

**Table 2:** Injection protocol recommendation for DICI-CT (non-evidence based, subject matter expert experiences).

Region	Cather Position	Injection Speed (mL/s)	Dilution (CM:saline)	Fluid Amount (mL)	Delay to Scan (s)
Abdominal Aorta	above diaphragm	10–15	1:5–10	120–180	5–10
CTPA	SMA	5–10	1:5	30	10–20
CTHA	Common hepatic artery	2–4	1:5–10	10–15	2–5
CTA lower extremities	above aortic bifurcation	5–10	1:5–10	120–150	5–7

**Table 3:** Non-evidence-based summary of differences between cone-beam CT (CBCT) and conventional CT (cCT) used typically in current hybrid Angio-CT systems.

	CBCT	cCT
Contrast resolution	5–10 HU	1 HU
Contrast dilution	1:1	1:5
Matrix size	2000 × 2000	512 × 512
Temporal resolution	low	high
CM Phase	single-double	multiple
Single slice imaging	more effort	less effort
Speed incl. prep	90 s	20 s
Breath hold	yes	no
Post-processing	more effort	less effort
Scan range—coverage	fixed	flexible up to 120 cm
FoV	max. 30 cm	∼50 cm
Isocentering	more effort	less effort
Dose	high	ca. 40% of CBCT [26]

## Abbreviations

CT	Computed Tomography
Angio	Angiography
MR	Magnetic Resonance Imaging
SPECT	Single Photon Emission CT
DSA	Digital Subtraction Angiography
OR	Operating Room
HCC	Hepatocellular Carcinoma
CPT	Current Procedural Terminology
QALY	Quality Adjusted Life Year
ICER	Incremental Cost Effectiveness Ratio
ceCT	Contrast-Enhanced CT
ceMR	Contrast-Enhanced MR
ceUS	Contrast-Enhanced Ultrasound
CM	Contrast Media
DICI-CT	Direct Intravascular Contrast Media Injection CT
EVAR	Endovascular Aortic Repair
CTA	CT-Angiography
CTP	CT-Perfusion
CTAP	CT Arterial Portography
CTHA	CT Hepatic Arteriography
CTDE	CT Dual Energy
SMA	Superior Mesenteric Artery
IO	Interventional Oncology
IR	Interventional Radiology
TACE	Transarterial Chemo Embolization
IQR	Inter-Quartile Range
CBCT	Cone-Beam CT
TIPS	Transjugular Intrahepatic Portosystemic Shunt
PAE	Prostate Artery Embolization
BPH	Benign Prostatic Hyperplasia
pT2EL	Persistent Type 2 Endoleaks
rAAA	Ruptured Abdominal Aortic Aneurysms
HER	Hybrid Emergency Room
